# Genomic Characterization of Radiation-Induced Intracranial Undifferentiated Pleomorphic Sarcoma

**DOI:** 10.1155/2021/5586072

**Published:** 2021-03-08

**Authors:** Christopher S. Hong, Edwin Partovi, James Clune, Anita Huttner, Henry S. Park, Sacit Bulent Omay

**Affiliations:** ^1^Department of Neurosurgery, Yale School of Medicine, New Haven 06511, CT, USA; ^2^Department of Pathology, Yale School of Medicine, New Haven 06511, CT, USA; ^3^Division of Plastic and Reconstructive Surgery, Department of Surgery, Yale School of Medicine, New Haven 06511, CT, USA; ^4^Department of Therapeutic Radiology, Yale School of Medicine, New Haven 06511, CT, USA

## Abstract

Intracranial undifferentiated pleomorphic sarcoma remains a rare pathology within the sarcoma literature that may arise primarily or secondary after radiation therapy. Despite first-line treatment with maximal surgical resection, followed by nonstandardized adjuvant chemotherapy/radiation regimens, clinical prognosis remains exceedingly poor. Furthermore, there is a lack of genetic or molecular characterization to guide potential for targeted therapies. We present genomic analysis of a radiation-induced intracranial undifferentiated pleomorphic sarcoma in an 83-year-old woman with notable KIT and PDGFRA alterations. Further similar genomic studies of intracranial pleomorphic sarcoma are needed to develop better therapies for this rare but challenging disease entity.

## 1. Introduction

Undifferentiated pleomorphic sarcomas (UPS) comprise a subset of malignant mesenchymal tumors, accounting for 15–20% of soft tissue sarcomas and typically affecting patients older than 50 years of age. [1] A subset of UPS arises as sequelae of prior radiation treatment, typically 9–12 years after initial radiotherapy. [2] Compared to systemic UPS, intracranial UPS has a worse prognosis with a mean overall survival of 1-2 years, which is perhaps even poorer in secondary UPS from prior radiation. [3, 4] The literature surrounding intracranial UPS, either arising primary or secondary to prior radiation, is limited to individual reports, detailing the clinical management of this rare but challenging disease entity. [4] However, to date, no studies have reported genetic or molecular characteristics of intracranial UPS. In this study, we present genomic data from a case of radiation-induced intracranial UPS and discuss the pertinent findings that define the molecular signatures of this lesion.

## 2. Case Presentation

### 2.1. Clinical Presentation

An 83-year-old woman presented with a growing solid right frontal scalp lesion over the past month that had not been present two months prior. Her past medical history was significant for diagnosis of a right frontal anaplastic astrocytoma, resected 18 years earlier, followed by adjuvant temozolomide and external radiation therapy (60 Gy in 30 fractions). She also underwent complete resection of stage IA lung adenocarcinoma four years prior. On examination, she was neurologically intact and cognitively at her baseline of mild dementia.

**Figure 1:**
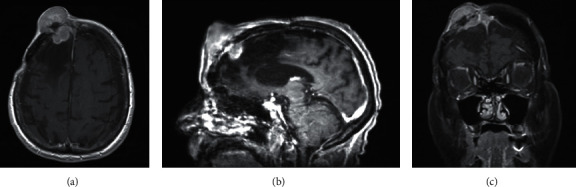
Preoperative MRI. Representative images from T1-weighted postcontrast MRI in the (a) axial, (b) sagittal, and (c) coronal orientations demonstrate a bilobed homogeneously enhancing lesion of the right frontal scalp with intracranial extension.

Magnetic resonance imaging (MRI) of her brain demonstrated a bilobed homogeneously enhancing right frontal scalp, measuring up to 4.5 cm, with 2.8 cm of intracranial extension (Figure 1). There were also expected encephalomalacic changes of the right frontal lobe with ex vacuo dilation of the frontal horn of the right lateral ventricle from prior astrocytoma resection. The differential diagnosis was felt to include a primary skin or soft tissue neoplasm, systemic metastasis, meningioma, or recurrence of a high-grade glioma with extracranial invasion. After consultation with neuro-oncology, surgical intervention was recommended to pursue a tissue-based diagnosis.

**Figure 2:**
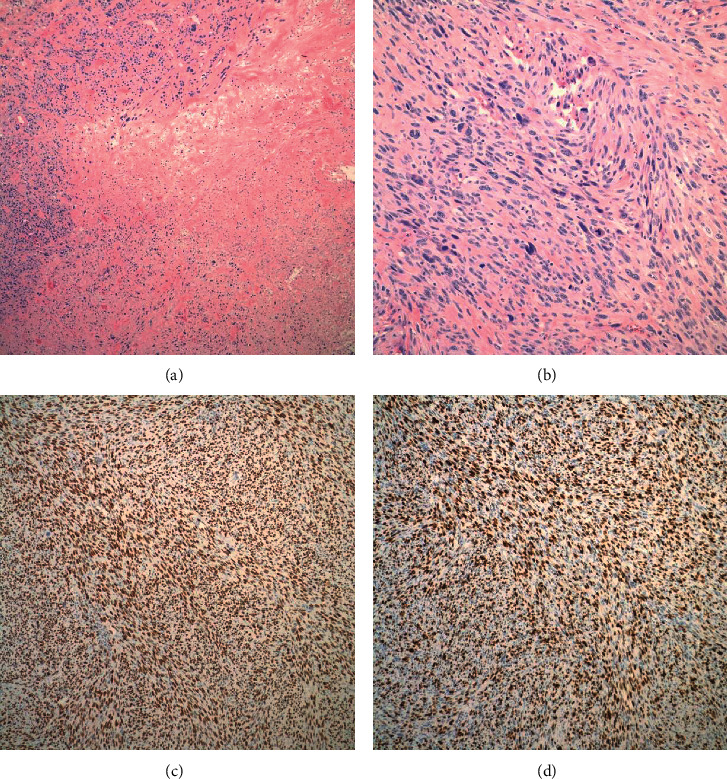
Histopathology. (a) Hematoxylin and eosin stain demonstrates a densely cellular spindle cell malignancy in partly fascicular or storiform pattern and areas of necrosis (100x magnification). (b) A higher magnification (200x) hematoxylin and eosin stain shows pleomorphic cells with a high mitotic rate, exceeding 20 mitoses per 10 high power fields. (c) The Ki-67 index is estimated at >75% (100x magnification). (d) Immunohistochemical staining for p53 nuclear expression is seen in >90% of cells (100x magnification).

The tumor was gross totally resected via a right frontal craniectomy with mesh cranioplasty to replace the bony defect, followed by complex scalp closure that involved rotation of a vascularized forehead flap and inset of a harvested split-thickness skin graft from the thigh. Pathologic review of the specimen revealed a densely cellular spindle cell malignancy composed of highly pleomorphic cells in a partly fascicular or storiform pattern with areas of necrosis (Figures 2(a) and 2(b)). The Ki-67 index was >75% (Figure 2(c)), and p53 nuclear expression was observed in >90% of cells (Figure 2(d)). Further immunohistochemical stains for vimentin were strongly positive, while those for S100, GFAP, EMA, and panCK were negative. Together these findings were suggestive of a high-grade pleomorphic sarcoma, French Federation of Cancer Centers Sarcoma Group (FNCLCC) grade 3. The patient recovered from surgery uneventfully. Her family declined further treatment, given her baseline poor performance status and age.

### 2.2. Genomic analysis

A targeted cancer gene panel (Oncomine Comprehensive Assay v3, Thermo Fisher Scientific, Waltham, MA, USA) was performed on the surgical specimen and peripheral blood, the latter serving as a normal, germline control specimen. This assay examines tumor DNA for mutations and/or amplifications in 146 cancer-related genes, as well as tumor RNA for the presence of gene fusion transcripts involving 44 oncogenic driver genes. This assay revealed somatic variants within the tumor, predicted to be deleterious based on SIFT [5] and PolyPhen [6] prediction algorithms, of KIT (V603D) and TP53 (Y220C), as well as 7 copy number amplifications of PDGFRA and KIT (Table 1).

**Figure 3:**
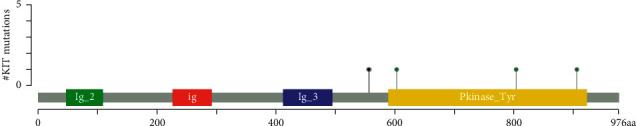
Schematic of KIT variants from the index patient and TCGA sarcoma database. The KIT V603D missense variant of the index patient is seen in the protein tyrosine kinase domain of the protein. The remaining four variants reported in three patients from the TCGA sarcoma database are plotted, including the frameshift variants (W557Gfs*∗*18 and Q556Rfs*∗*8) in a case of leiomyosarcoma, the R804Q missense variant in a separate case of leiomyosarcoma, and the C906R missense variant in a case of malignant peripheral nerve sheath tumor.

These results were cross-referenced with The Cancer Genome Atlas (TCGA) sarcoma cohort [7], comprising 206 samples, including 44 cases of UPS. There were four total KIT variants predicted to be deleterious in this database in three patients, comprising two cases of leiomyosarcomas (patient 1: R804Q; patient 2: W557Gfs*∗*18, Q556Rfs*∗*8) and one malignant peripheral nerve sheath tumor (C906R). The KIT V603D mutation observed in our patient was located in the protein tyrosine kinase domain and plotted alongside data from the TCGA database with MutationMapper [8, 9] (Figure 3). TP53 mutations were reported in 69 (33.5%) patients in the TCGA sarcoma database, including 18 (26.1%) cases of UPS. Regarding copy number alterations, the TCGA database reported three cases of sarcomas with copy number amplifications in KIT, comprising two UPS and one myxofibrosarcoma. Notably, these three comprised the majority of the four total cases in the database that harbored PDGFRA copy number amplification with the remaining case also being UPS.

## 3. Discussion

Intracranial UPS remains a rare entity within the sarcoma literature. First-line treatment is maximally safe surgical resection. Postoperative chemotherapy and/or radiation have been reported in cases, but there remains no histopathological or molecular framework in which to guide adjuvant therapy [10, 11]. Most recently, Wapshott et al. provided a comprehensive review of the literature of intracranial UPS detailing the clinical characteristics and management of reported patients but concluded a lack of molecular characterization of this disease entity to guide clinical prognostication and targeted therapies [4].

Given sarcomas as a whole represent a highly heterogeneous group of cancers, a diverse array of oncogenic mutations and/or copy number changes has been described [12]. However, the distinct pathogenesis of radiation-induced sarcomas, compared to their de novo counterparts, remains understudied. Limited reports have suggested that mutations in RB1 and TP53 [13–15] and amplifications of MYC may be more prevalent in radiation-induced sarcomas [16, 17]. Likewise, multiomics studies have associated transcriptomic signatures associated with increased oxidative stress due to mitochondrial dysfunction [18] and driver oncogenic events, characterized by excesses of genome-wide deletions and balanced inversions [19]. Our results overlapped with some of these existing data, discussed as follows.

Somatic activating mutations in KIT and PDGFRA have been well-described and are mutually exclusive in gastrointestinal stromal tumors [20, 21], leading to the approval of the tyrosine kinase inhibitors, imatinib, sunitinib, and regorafenib for these rare tumors [22]. Activating KIT mutations are also prevalent in a subset of melanoma, and notably, imatinib, sunitinib, dasatinib, and nilotinib have demonstrated efficacy in these patients, particularly those with the L576P and K642E variants [23]. Among the KIT variants reported in the TCGA sarcoma database, none had sufficient preclinical data of the underlying mechanisms to guide adjuvant therapy. In our patient, the KIT V603D missense variant was predicted to be deleterious, residing within the protein tyrosine kinase domain. However, while this variant has been reported in a squamous cell carcinoma of the lung in the COSMIC database [24], its downstream effects have not been studied in vitro, and therefore, the potential for tyrosine kinase therapy against this variant also remains unclear. Notably, we also detected copy number amplifications of this KIT variant, as well as in wildtype PDGFRA. Concurrent copy number changes in these genes commonly occur, given both genes are located on chromosome 4q12 [25]. While simultaneous mutations in KIT and PDGFRA have been reported in some soft tissue sarcomas [26], copy number gains of both genes secondary to chromosome 4q12 amplification have been reported across the cancer landscape [25], including the two cases referenced from the TCGA sarcoma database. Notably, early clinical data have suggested a role for empiric tyrosine kinase inhibitor therapies, specifically imatinib, pazopanib, and axitinib in patients with chromosome 4q12 amplifications, involving KIT and PDGFRA [25].

We also detected a missense variant of the tumor suppressor TP53, predicted to be deleterious and located in a previously reported hotspot domain. This variant has been previously reported to be a recurrent somatic mutation in cancer, as well as a pathogenic variant associated with Li–Fraumeni syndrome. Loss of TP53, despite its prominence across many cancers, is relatively under-represented among sarcomas [27]. Despite a relatively lower prevalence among the cases of UPS in the TCGA cohort, other studies have reported upwards of two-thirds of extracranial, systemic cases exhibiting TP53 mutations [28, 29]. Interestingly, the majority retains one wildtype TP53 allele [28], but additional losses in p14ARF, a protein product of the CDKN2A gene and implicated in proteasomal degradation of p53 via interaction with MDM2 [30], may be required for direct TP53 inactivation [28]. While our comprehensive genetic panel did not assess p14ARF status, it did not detect any alterations in CDKN2A, suggesting that heterozygous loss of TP53 function cannot be definitely attributed to pathogenesis in our patient's case.

Taken together, this study reports novel genomic findings of intracranial UPS, which remains severely lacking in molecular characterization to guide clinical decision-making. While further genetic reports are necessary, our findings suggest potential actionable targets against KIT and PDGFRA alterations that represent promising therapeutic interventions for this rare but challenging disease entity.

## Figures and Tables

**Table 1:** Results from the targeted cancer gene panel of the index patient.

Gene	Gene full name	Consequence	Variant(protein)	Variant (coding DNA)	Predicted effects	Allelic fraction	Chromosome	Copy number
KIT	v-kit Hardy-Zuckerman 4 feline sarcoma viral oncogene homolog	Missense	V603D	c.1808T > A	Deleterious (SIFT), probably damaging (PolyPhen)	0.73	4q12	7
TP53	Tumor protein p53	Missense	Y220C	c.659A > G	Deleterious (SIFT), probably damaging (PolyPhen)	0.62	17p13	1
PDGFRA	Platelet-derived growth factor receptor, alpha polypeptide	N/A	N/A	N/A	N/A	N/A	4q12	7

## Data Availability

The data analyzed in the current work are available from the corresponding author on reasonable request
